# Small Molecule DFPM Derivative-Activated Plant Resistance Protein Signaling in Roots Is Unaffected by EDS1 Subcellular Targeting Signal and Chemical Genetic Isolation of *victr* R-Protein Mutants

**DOI:** 10.1371/journal.pone.0155937

**Published:** 2016-05-24

**Authors:** Hans-Henning Kunz, Jiyoung Park, Emily Mevers, Ana V. García, Samantha Highhouse, William H. Gerwick, Jane E. Parker, Julian I. Schroeder

**Affiliations:** 1 Division of Biological Sciences, Section of Cell and Developmental Biology, University of California San Diego, La Jolla, California 92093–0116, United States of America; 2 Center for Marine Biotechnology and Biomedicine, Scripps Institution of Oceanography, University of California San Diego, La Jolla, California, 92093–0212, United States of America; 3 Max-Planck Institute for Plant Breeding Research, Department of Plant-Microbe Interactions, D-50829 Cologne, Germany; 4 Skaggs School of Pharmacy and Pharmaceutical Sciences, University of California San Diego, La Jolla, California, 92093, United States of America; Ghent University, BELGIUM

## Abstract

The small molecule DFPM ([5-(3,4-dichlorophenyl)furan-2-yl]-piperidine-1-ylmethanethione) was recently shown to trigger signal transduction via early effector-triggered immunity signaling genes including *EDS1* and *PAD4* in *Arabidopsis thaliana* accession Col-0. Chemical genetic analyses of *A*. *thaliana* natural variants identified the plant Resistance protein-like Toll/Interleukin1 Receptor (TIR)-Nucleotide Binding (NB)-Leucine-Rich Repeat (LRR) protein VICTR as required for DFPM-mediated root growth arrest. Here a chemical genetic screen for mutants which disrupt DFPM-mediated root growth arrest in the Col-0 accession identified new mutant alleles of the TIR-NB-LRR gene *VICTR*. One allele, *victr-6*, carries a Gly216-to-Asp mutation in the Walker A domain supporting an important function of the VICTR nucleotide binding domain in DFPM responses consistent with VICTR acting as a canonical Resistance protein. The essential nucleo-cytoplasmic regulator of TIR-NB-LRR-mediated effector-triggered immunity, EDS1, was reported to have both nuclear and cytoplasmic actions in pathogen resistance. DFPM was used to investigate the requirements for subcellular EDS1 localization in DFPM-mediated root growth arrest. EDS1-YFP fusions engineered to localize mainly in the cytoplasm or the nucleus by tagging with a nuclear export signal (NES) or a nuclear localization signal (NLS), respectively, were tested. We found that wild-type EDS1-YFP and both the NES and NLS-tagged EDS1 variants were induced by DFPM treatments and fully complemented *eds1* mutant plants in root responses to DFPM, suggesting that enrichment of EDS1 in either compartment could confer DFPM-mediated root growth arrest. We further found that a light and O_2_-dependent modification of DFPM is necessary to mediate DFPM signaling in roots. Chemical analyses including Liquid Chromatography-Mass Spectrometry and High-Resolution Atmospheric Pressure Chemical Ionization Mass Spectrometry identified a DFPM modification product that is likely responsible for bioactivity mediating root growth arrest. We propose a chemical structure of this product and a possible reaction mechanism for DFPM modification.

## Introduction

In many organisms, the screening of chemical libraries has been used successfully to identify inhibitors or agonist molecules [1]. Newly isolated compounds are powerful tools for overcoming genetic functional redundancy or mutant lethality and therefore help to characterize mechanisms underlying gene networks [2]. The pathogen response in plants involves a complex defense signaling network. Nucleo-cytoplasmic proteins EDS1 and PAD4 are key players in basal and effector-triggered immunity (ETI) by controlling transcriptional reprogramming of defense pathways [3–6]. Both loci were discovered through classic forward genetic screens of *Arabidopsis thaliana* mutants treated with pathogens, eg. *Hyaloperonospora arabidopsidis* (formerly *Peronospora parasitica*) for *eds1* [7] and *Pseudomonas syringae* for *pad4* [8]. In both cases, mutant lines showed increased disease susceptibility.

Processes operating upstream of EDS1 and PAD4 are more variable. In *Arabidopsis thaliana*, ~ 150 *R**esistance* (*R*)-genes encode sensors of a wide range of pathogen effectors which are delivered by the pathogen into plant host cells to disable basal immunity [9, 10]. Based on their N-termini, R-proteins can be further separated into coiled-coil (CC)-nucleotide binding (NB)-leucine-rich repeat (LRR) and Toll/Interleukin1 Receptor (TIR)-NB-LRR proteins [10, 11]. Sequence variations between *R*-genes are utilized by plants to respond, directly or indirectly, to diverse pathogen effectors. Therefore *R*-genes are the most variable plant gene family [12, 13]. Using a chemical genetics approach, a small molecule named DFPM ([5-(3,4-dichlorophenyl)furan-2-yl]-piperidine-1-ylmethanethione) was isolated which triggers an accession-specific fast immune response in *Arabidopsis thaliana* Col-0 [14, 15]. Within a few hours of DFPM exposure, strong primary root growth arrest is observed [15]. This response relies on a locus that exhibits natural variation among Arabidopsis accessions and encodes a TIR-NB-LRR protein designated VICTR (Variation in compound triggered root growth response) [15]. The *VICTR* gene is encoded in tandem with its closest homolog *VICTR Like 1* (*VICTL1*). Notably, a loss-of-function mutation in *VICTL1* does not compromise DFPM-mediated root growth arrest [15].

The function of most NB-LRR proteins depends on ATP/ADP or GTP/GDP binding and hydrolysis at a conserved nucleotide binding site [10]. It remains unclear whether VICTR acts as a canonical R-protein requiring a functional nucleotide-binding site, as only T-DNA insertion mutants were available so far for analyses. Initial evidence that VICTR might be part of an ETI signaling pathway stems from the genetic requirement of *EDS1* and *PAD4* as well as co-chaperone encoding genes *RAR1* and *SGT1b* in response to the small molecule DFPM [14, 15].

Arabidopsis EDS1 and PAD4 are nucleo-cytoplasmic proteins [6]. Nuclear localization of EDS1 protein was found to be necessary for transcriptional defense reprogramming and effective pathogen resistance in leaves [16, 17]. Also, a role for the EDS1 cytoplasmic pool was suggested based on resistance phenotypes of mis-localized EDS1 fused to a nuclear export sequence (NES) or held in the cytoplasm by a glucocorticoid hormone-binding (HBD) domain [17]. *eds1* and *pad4* mutants exhibited a similar degree of insensitivity to DFPM as *victr* mutants in root growth arrest assays [14, 15]. Therefore, DFPM-triggered root growth arrest produces a facile and powerful read-out to screen for new mutants in TIR-NB-LRR signaling pathways. These features also offer the possibility to use the DFPM-triggered root growth arrest to further interrogate the importance of EDS1 subcellular localization in the DFPM-mediated signal transduction process.

DFPM or DFPM-generated molecules appear to activate the TIR-NB-LRR protein VICTR in a very specific manner because a number of related DFPM derivatives were tested revealing that only small changes in the molecular structure or side groups significantly diminished bioactivity of DFPM [14, 15]. Most molecules from commercial chemical libraries are dissolved in dimethyl sulfoxide (DMSO) and show relatively poor solubility in aqueous solutions. Due to their lipophilicity, this has the advantage that molecules can diffuse into cells via the plasma membrane. However, candidate molecules can undergo reactions with a solvent or other substances inside cells, and therefore it is important to characterize the chemical characteristics of each bioactive compound individually. Here, we show that a modified product of DFPM, rather than DFPM itself, is the likely bioactive molecule in DFPM-mediated root growth arrest and we provide information on its chemical properties.

In this report, using a DFPM-mediated root growth arrest screen, we identify important residues within the VICTR protein for R-protein signaling. We establish that the DFPM root growth response is equally activated by EDS1 pools enriched in the nucleus or the cytoplasm. To obtain further insights into optimal handling for this potent chemical genetic screening, we also investigate DFPM stability and propose a putative bioactive product derived from DFPM.

## Results

### Chemical genetic screening identifies new mutations in *VICTR*

**Fig 1:**
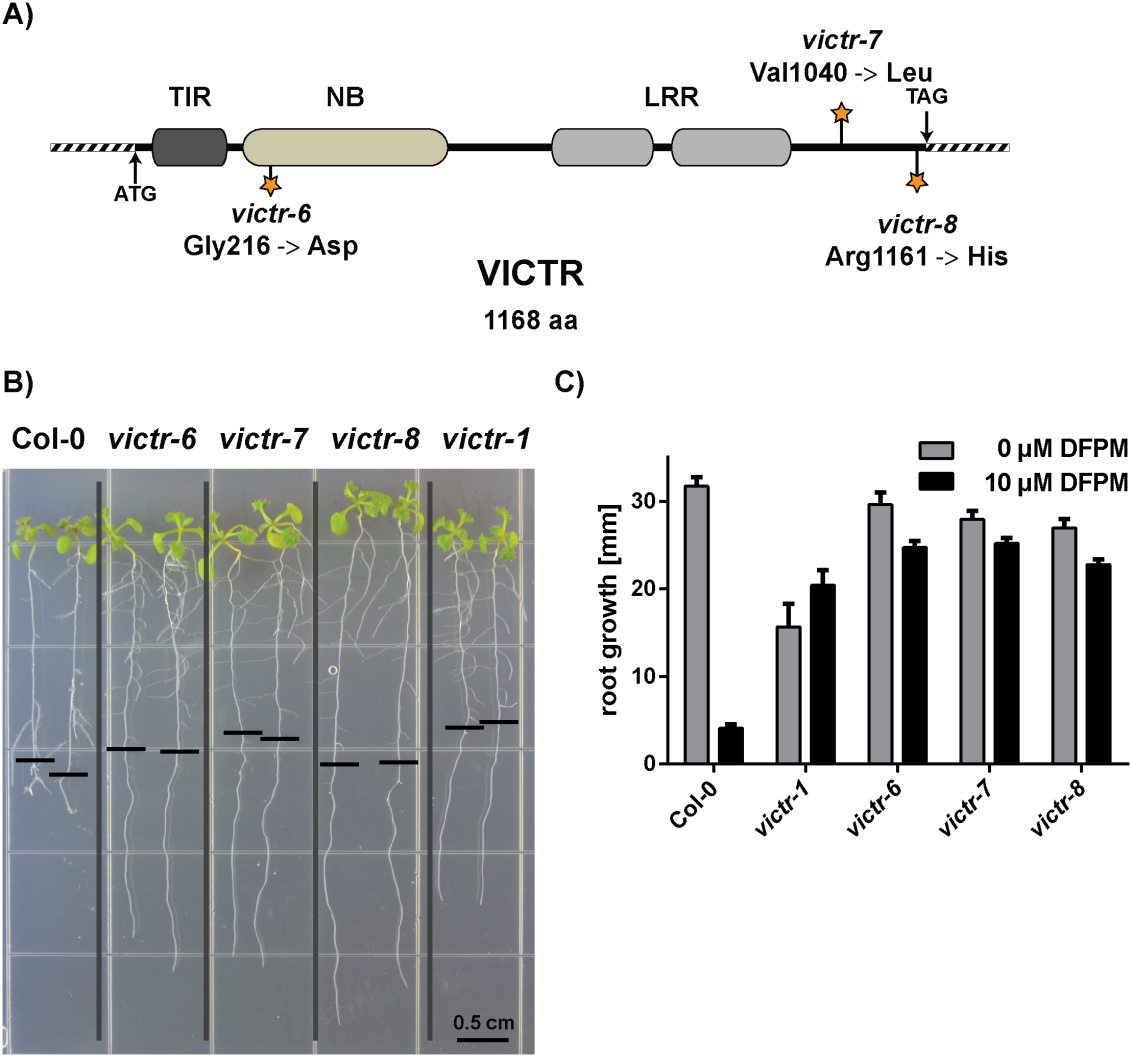
Mapping of new *victr* mutant alleles isolated via facile DFPM-mediated root growth arrest. A) Overview and amino acid changes (marked with stars) in three newly isolated EMS mutants in the *VICTR* locus. B) EMS point mutants in *VICTR* (Col-0), *victr-6*, *victr-7 and victr-8*, showed insensitivity to DFPM similarly to T-DNA mutant *victr-1* whereas the wild-type Col-0 control was fully arrested in root growth assays. The black horizontal bars mark the root tip position when plants were exposed to 10 μM DFPM. The vertical grey bars separate the indicated genotypes grown on the same plate for clarity. C) Quantitative analyses of root growth assays shown in B). With 10 μM DFPM all three EMS *victr* point mutants and *victr-1* showed a similar DFPM insensitivity compared to the their growth in 0 μM DFPM. Wild type Col-0 showed a strong inhibition in root growth upon DFPM treatment. Error bars represent SEM (n = 7–18 seedlings for 10 μM DFPM, n = 3–17 for 0 μM DFPM).

Natural variation in the *VICTR* locus and T-DNA insertion mutants were used initially to identify and characterize functions of VICTR [15]. Although T-DNA insertion lines represent a powerful tool to study full gene loss-of-function defects, they rarely contribute to a better understanding of the protein structure. Approximately 10,000 M2 individuals from 24 different parental groups of an EMS mutagenized population (Col-0 accession) were screened using the DFPM-mediated root growth arrest as a readout. In the initial screen, 38 DFPM insensitive individuals were isolated. The identified DFPM insensitive mutants were backcrossed to the *victr-1* T-DNA insertion line [15] and subsequently investigated for allelism in the F1 generation using the DFPM-induced root growth arrest assay. Three mutants that fell into the same *VICTR*-linked complementation group were backcrossed to Col-0 wild-type plants and investigated in detail by sequencing the entire *VICTR* locus of the resulting F2 individuals to test for functionally crucial amino acids in VICTR. As shown in a VICTR protein schematic (Fig 1A), three independent point mutations in the *VICTR* gene were identified and characterized. The first EMS mutant *victr-6* carries a glycine 216 to aspartic acid mutation in the conserved nucleotide-binding site of the VICTR TIR-NB-LRR protein. Gly 216 is part of the Walker A motif, or P-loop, that has the general consensus sequence *GX*_*4*_*GK*[*T/S*] [18]. Since the adjacent lysine residue in the Walker A motif is needed to coordinate *β*- and *γ*-phosphates of the bound NTP and indispensible for activation of Resistance proteins [10, 18, 19], the loss of this highly conserved glycine in *victr-6* is predicted to diminish ADP/ATP binding to the NB domain and hydrolysis.

In *victr-7* and *victr-8*, respectively, the mutations were in a non-conserved portion of the protein C-terminal to the LRR domain (Fig 1A). The *victr-7* and *victr-8* mutations were located at two of the few positions which vary between VICTR and its closest homologue, VICTL1. In *victr-7*, valine 1040 is replaced by leucine, which is the amino acid at the same position in VICTL1. The third EMS mutagenized line, *victr-8* had a mutation close to the 3’ end of the *VICTR* coding sequence. This mutation results in an arginine 1161 to histidine exchange (Fig 1A).

### DFPM signaling is unaffected by EDS1 subcellular targeting signals

In our earlier studies, we documented protein-protein interaction of VICTR with EDS1 in the nucleus and that DFPM-triggered VICTR-mediated root growth arrest requires *EDS1* function [15]. In line with this finding, *eds1-2* mutant plants exhibit DFPM insensitivity in roots and leaves [14, 15]. To expand on these results showing interdependency, we tested *VICTR* gene expression in *eds1-2* mutants compared to wild-type controls (S1A Fig). Interestingly, we found that full induction of *VICTR* gene expression by DFPM requires *EDS1*. However, significant residual *VICTR* transcript induction was detectable in *eds1-2* mutants indicating that *VICTR* induction does not exclusively depend on *EDS1* (S1A Fig).

EDS1 is a nucleo-cytoplasmic protein [6]. Experiments employing the *Pseudomonas syringae* bacterial pathogen as a trigger of *EDS1*-dependent ETI or basal immunity suggested that nucleo-cytoplasmic trafficking through the nuclear pore complex is important for full pathogen resistance in leaves [17, 20]. We utilized *eds1*-*2* transgenic lines (in Col-0 accession) NES #2–11 and NES #3–4 that express functional EDS1-YFP fused to a C-terminal nuclear export sequence (EDS1-YFP-NES) [17]. This fusion caused EDS1 to accumulate mainly in the cytoplasm of leaf cells and produced intermediate pathogen resistance in leaves [17]. As controls, a line expressing EDS1-YFP alone (EDS1-YFP) or EDS1-YFP fused to a mutated, inactive NES (EDS1-YFP-nes) (nes #1–2) were also tested. These conferred full pathogen resistance in leaf assays [17]. In a complementary approach, *eds1-2* transgenic lines NLS #A5 and NLS #B2 expressing high and low amounts, respectively, of EDS1-YFP fused to a C-terminal SV40 nuclear localization signal (NLS) which targets EDS1 to the nucleus [21], were examined. All transgenic EDS1-YFP constructs were expressed under the native EDS1 promoter [17, 21]. With these transgenic materials, we investigated whether cytoplasmic or nuclear enrichment of EDS1-YFP alters the efficiency of the DFPM-triggered root growth arrest response.

**Fig 2:**
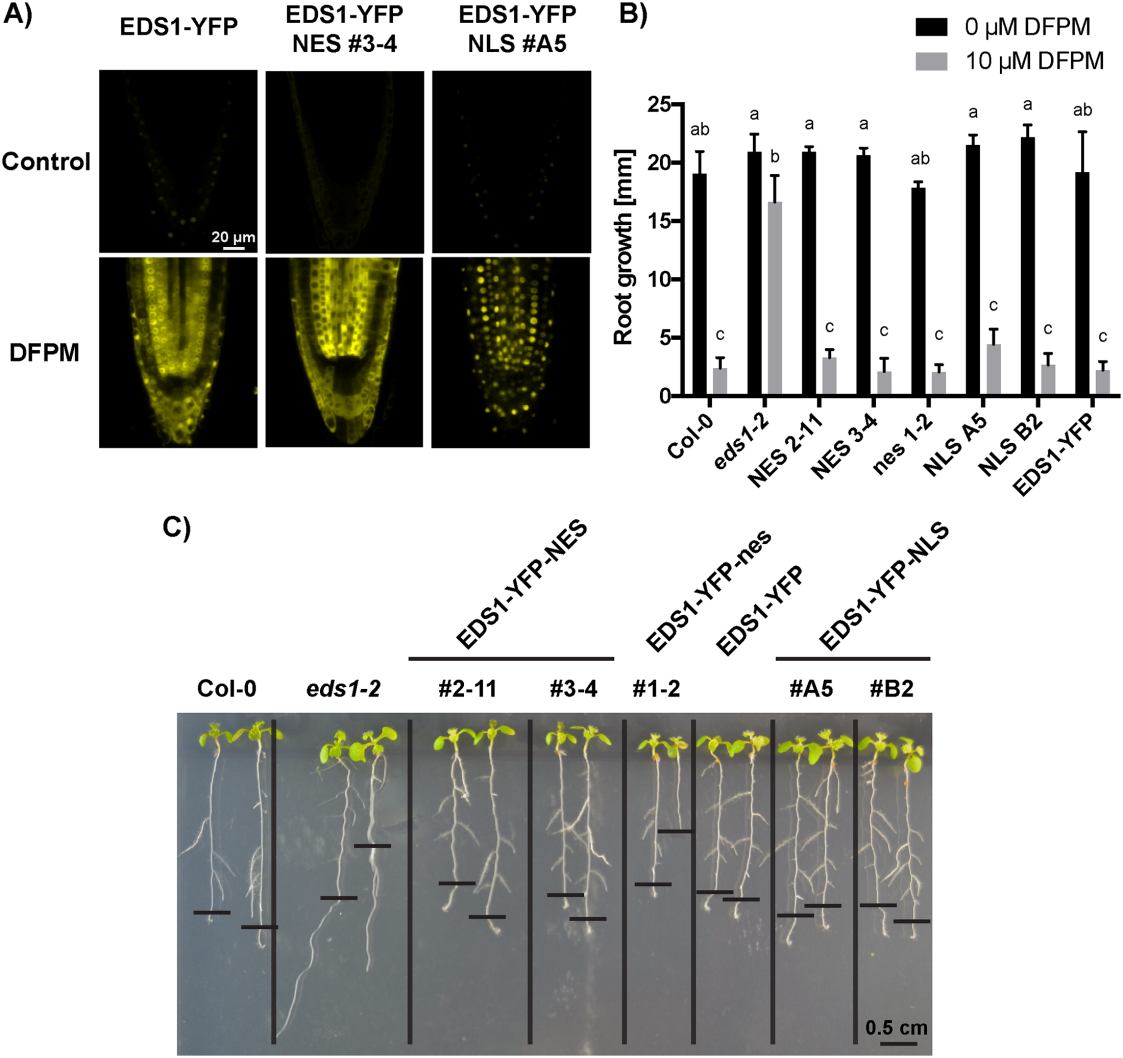
DFPM-induced root growth arrest is unaffected by the subcellular targeting signals of EDS1-YFP protein. A) EDS1-YFP localization in the root of EDS1-YFP, EDS1-YFP-NES and EDS1-YFP-NLS in *eds1-2*. All transgenic EDS1-YFP constructs were driven by the native EDS1 promoter. Interestingly, EDS1-YFP signals increased after 24 hours of 10 μM DFPM application compared to the non-treated controls. Scale bar applies to all 6 images. Confocal gain and pinhole parameters were identical in all six images. At least 3 plants were observed for DFPM treated condition. B-C) Both NES- or NLS-tagged EDS1 protein versions expressed in *eds1-2* were capable of complementing the *eds1-2* phenotype in DFPM-mediated primary root growth arrest. Seven day-old seedlings were exposed to 10 μM DFPM. Three days after treatment, two independent lines of EDS1-YFP-NES and EDS1-YFP-NLS showed the same DFPM sensitivity as wild-type Col-0 and EDS1-YFP control (B, C). A mutated control EDS1-YFP-nes also showed a similar sensitivity to DFPM. Means with different letters are grouped based on two-way ANOVA and Tukey’s test, *P* < 0.05). Error bars represent SD (n = 8 to 12 seedlings per condition for 10 μM DFPM, n = 2 to 4 for 0 μM DFPM). Representative plants are shown in (C). The black horizontal bars mark the root tip position when plants were exposed to DFPM. The vertical black bars separate the indicated genotypes grown on the same plate for clarity.

Localization of EDS1-YFP mainly to the cytoplasm (in lines NES #3–4 and NES #2–11) or the nucleus (in lines NLS #A5 and NLS #B2) was observed previously in confocal live-cell fluorescence imaging and biochemical fractionation experiments performed on leaf tissues [17, 21]. Because DFPM triggers growth arrest in roots, we performed fluorescence imaging of root cells to verify the subcellular localization of the EDS1 fusion constructs. Notably, a fluorescence signal for EDS1-YFP was barely detectable in untreated roots (Fig 2A, control). Application of 10 μM DFPM for 24 h led to a strong increase in the fluorescence signal in roots including meristematic and elongation zones (Fig 2A, DFPM). This observation is consistent with *EDS1* transcript accumulation upon DFPM exposure found in whole seelings [14] and the same DFPM-induction was seen in all tested EDS1-YFP mislocalized lines (Fig 2A, S2 Fig). To verify the fluorescence-based results on DFPM-induced *EDS1* expression in root tissues, we carried out qPCR analyses and confirmed that *EDS1* gene transcription is increased by DFPM treatments in a dose-dependent fashion (S1B Fig). *EDS1* was also found to be upregulated in shoot tissues in response to DFPM (S1B Fig).

Wild-type EDS1-YFP localized in the cytoplasm and the nucleus of root cells (Fig 2A). By contrast, EDS1-YFP in line NES #3–4 exhibited YFP signals predominatly in the cytoplasm (Fig 2A). A clear reduction in nuclear YFP fluorescence was observed in nuclei from root cells including the root meristemic zone (Fig 2A), elongation zone and differentiation zone of NES #3–4. An opposite pattern was observed for EDS1-YFP in NLS lines #A5 and #B2 in which fluorescence was detected in nuclei of root cells (Fig 2A, S2 Fig). As expected, the control line EDS1-YFP-nes #1–2 exhibited YFP signals in the cytoplasm and the nucleus of root cells (S2 Fig). Together, these findings suggest that subcellular localization of DFPM-induced EDS1-YFP can be modulated in roots.

We found that both NES-tagged and NLS-tagged EDS1-YFP proteins fully complemented the *eds1-2* mutant in response to DFPM (Fig 2B and 2C). In EDS1-YFP-NES lines #2–11 and #3–4, DFPM-mediated root growth arrest responses were equivalent to those of wild-type Col-0 and EDS1-YFP, showing significant root growth arrest at 3 d after exposure to 10 μM DFPM (Fig 2B and 2C). The EDS1-YFP-nes #1–2 control line also exhibited root growth arrest in response to DFPM treatment. As observed in earlier studies, the *eds1-2* mutant without *EDS1* transgenes was insensitive to DFPM treatment [14, 15] (Fig 2B and 2C). EDS1-YFP-NLS line #A5 (a high EDS1-YFP expressor) and #B2 (low EDS1-YFP expressor) [21] also showed a wild-type like response (Fig 2B and 2C). We concluded that EDS1 can trigger effectively DFPM-triggered root growth arrest when directed to the cytoplasmic or nuclear compartments of root cells.

### DFPM decreases root cell viability in accession Col-0

**Fig 3:**
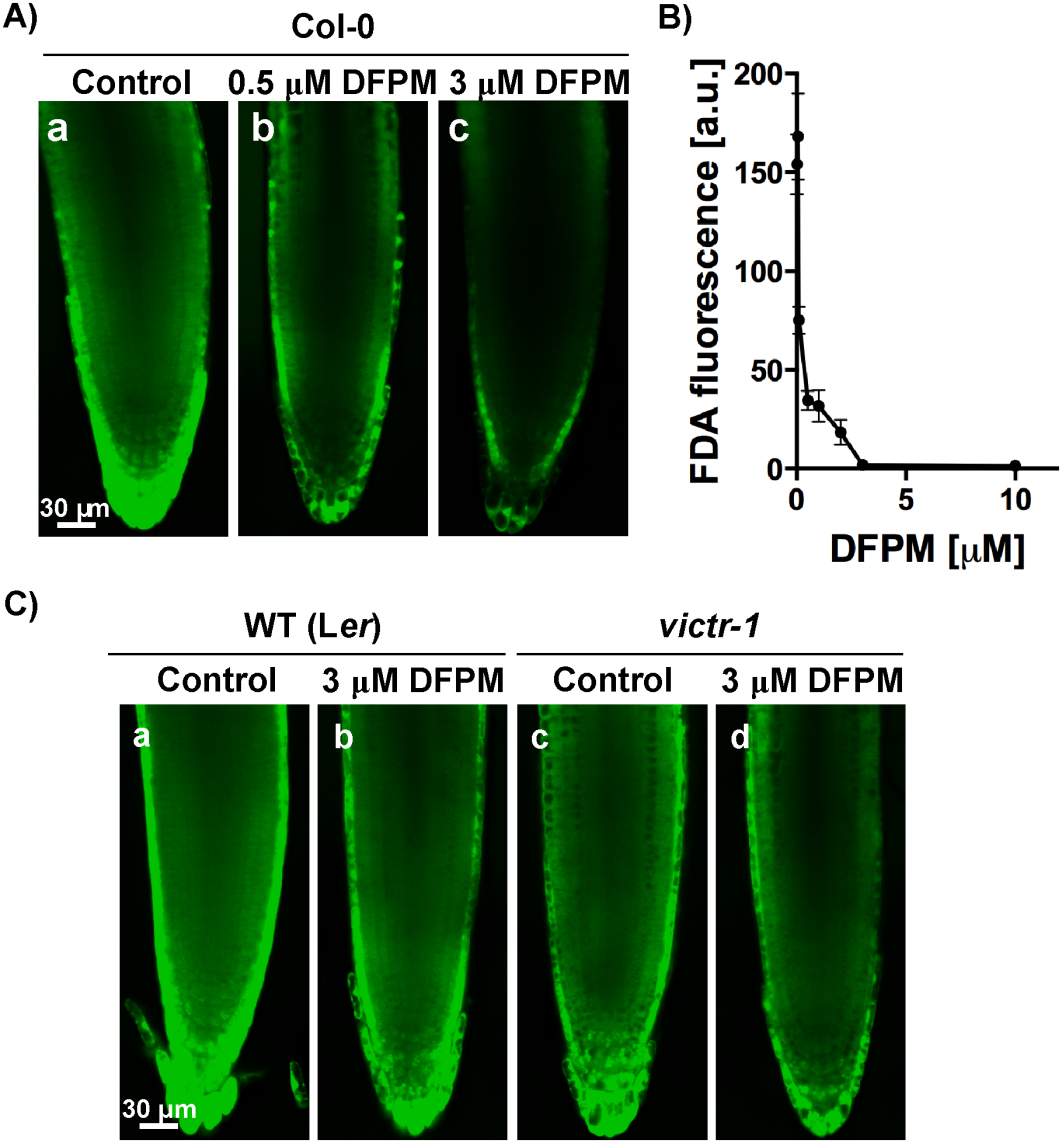
DFPM treated-wild type (Col-0) seedlings exhibit decreased cell viability in roots. A) Root cell viability of Col-0 root tissues decreased as concentrations of DFPM increased. Wild-type (Col-0) seedlings were exposed to the indicated concentrations of DFPM or DMSO (control) for 24 hours and cell viability was visualized by staining with fluorescein diacetate (FDA) dye. B) Fluorescence intensity of FDA was measured in root tips exposed to different concentrations of DFPM from 0.5 to 10 μM. (n = 3–11 per condition. error bars represent s.e.m.) C) Roots of the DFPM-insensitive L*er* accession [15] and *victr-1* mutant (Col-0) seedlings exhibited a marginal decrease in cell viability staining after exposure to 3 μM DFPM for 24 hours. Constant gain and pinhole parameters were used for all images.

Previously, DFPM was shown to cause a rapid root growth arrest of primary roots in Col-0 wild-type plants [14, 15]. This effect is dependent on *EDS1* and *PAD4*, which are also needed to regulate cell death at pathogen infection sites [17, 22, 23]. To obtain further insights into the DFPM-mediated root growth arrest, root cell viability of DFPM-treated tissues was investigated using fluorescein diacetate (FDA) staining [24]. The green fluorescence signal of fluorescein, an FDA metabolite, is a positive indicator for cell viability. When 10 day-old Col-0 wild-type seedlings were exposed to DFPM-containing media with increasing concentrations from 0.5 to 10 μM for 24 hours, FDA fluorescence signals from root tip tissues decreased in a DFPM concentration-dependent manner compared to non-treated controls (Fig 3A and 3B). DFPM concentrations above 3 μM led to a strong FDA fluorescence signal reduction (IC_50_ = ~ 0.378 μM; Fig 3B).

*VICTR* is necessary and sufficient for DFPM-triggered primary root growth arrest [15]. Because of the absence of a functional *VICTR* allele in the Landsberg *erecta* (L*er*) accession, L*er* accession seedlings showed DFPM insensitivity to a similar extent to the *victr-1* T-DNA insertion mutant [15]. L*er* seedlings exposed to 3 μM DFPM exhibited a marginal decrease in FDA signal compared to mock treated wild-type L*er* control (Fig 3C). Similarly, in *victr-1* FDA signals were mildly reduced after exposure to 3 μM DFPM (Fig 3C). These results suggest that in the presence of a functional *VICTR* locus, DFPM application causes reduced cell viability which correlates with DFPM-induced root growth arrest.

### DFPM is light sensitive in aqueous solutions

**Fig 4:**
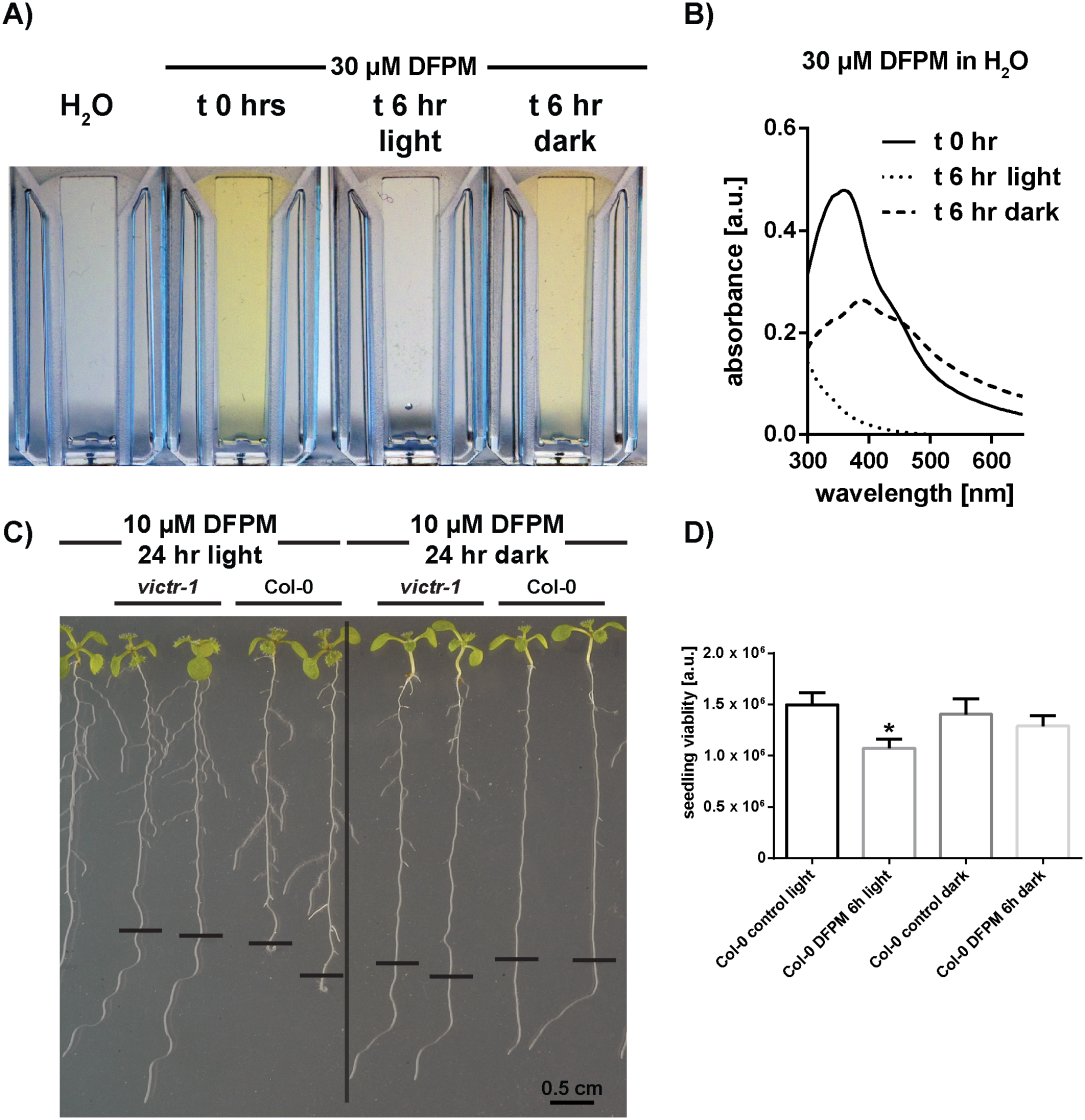
Light-induced DFPM degradation in aqueous solutions and its impact on bioactivity. A) After 6 hours in the light a clear loss of the yellow color can be detected in an aqueous 30 μM DFPM solution. B) UV-Vis absorbance scans of 30 μM DFPM solution. The fresh solution exhibits a characteristic maximum peak at around 360 nm. This peak was absent if the solution was kept in the light for 6 hours. Less pronounced decay was observed if the solution was protected from light. C) The bioactivity of DFPM is dependent on the presence of light. No growth arrest was found if roots were treated for 24 hours with DFPM in the dark (right), while Col-0 wild type plants treated with DFPM in the presence of light exhibited root growth arrest (left). After 24 hours of DFPM treatment seedlings were retransferred to 1/2 MS without DFPM to recover, and 3 days after retransfer the image was taken. Black horizontal bars indicate the primary root lengths at the time of retransfer. The black vertical bar separates two groups with different light treatments prior to retransferring to the same plate for recovery. D) FDA staining of 1 week-old seedlings treated with 15 μM DFPM for 6 hours. Cell viability was significantly reduced, only if light was present in combination with DFPM. Asterisk stands for *P* < 0.0001.

Similar to many other compounds from commercial chemical libraries, DFPM represents a colored, sparingly aqueous soluble, nonpolar substance (https://scifinder.cas.org) [14] that is solubilized in a DMSO stock solution. Research on storing conditions of screening compounds has revealed that prolonged storage of organic substances even in DMSO may result in a partial chemical degradation of the substance [25]. This effect is mainly due to DMSO slowly reacting with certain classes of chemical compounds or slowly decomposing in the presence of water, organic or inorganic acids, and strong oxidizing agents [25]. Inevitably, these effects are not limited to the storage process but might also occur during bioassays when the DMSO-dissolved screening compounds are mixed with aqueous solutions. At concentrations higher than 30 μM in aqueous buffers, DFPM is insoluble and can be detected by the occurrence of scatter and yellow cloudiness in the dilution. Moreover, in analyses of the stability of DFPM, we found that after 6 hours at room temperature, our freshly prepared working dilutions in water lost their typical yellow color (Fig 4A and 4B). Interestingly, if an aliquot from the same original dilution was protected from light a less-pronounced loss of color was detectable visually (Fig 4A).

This observation was confirmed by ultraviolet-visible spectral (UV-Vis) scans (Fig 4B). In the case of a fresh 30 μM DFPM solution a characteristic maximum absorbance peak at around 360 nm was detected. If the solution was exposed to light, this peak disappeared and the solution turned colorless. Less pronounced decrease in the absorbance and a broader peak were measured for dark-stored samples (Fig 4B).

To test whether the light and solvent dependent conversion has an impact on the bioactivity of DFPM, independent experimental procedures were designed. First, a rescue of root growth arrest assay was performed. As shown earlier, a temporary exposure of Col-0 wild-type seedlings to 10 μM DFPM for as little as 8 hours is sufficient to induce a complete root growth arrest [15]. Intriguingly, if plants were treated with 10 μM DFPM in the dark for 24 h, wild-type roots did not show a root growth arrest (Fig 4C, right). Thus after rescue of seedlings to a ½ MS plate without DFPM, wild-type roots that had been exposed to DFPM in the dark continued to grow and were indistinguishable from DFPM-insensitive *victr* mutant plants. However, if plants were exposed to DFPM in the same condition, but in the presence of light, a significant root growth arrest was observed in wild-type Col-0 (Fig 4C, left).

In addition, a 96 well plate reader assay was established to quantify the DFPM-induced decrease in cell viability. For this experiment 7 day-old Col-0 seedlings were treated with 15 μM DFPM for 6 hours either in the presence or absence of light. Afterwards, an FDA staining was performed and fluorescence signals were analyzed. As shown in Fig 4D, only when DFPM was applied in combination with light a clear decrease in cell viability was detected. The cell viability in the presence of light was also lower compared to DFPM incubation in the dark.

**Fig 5:**
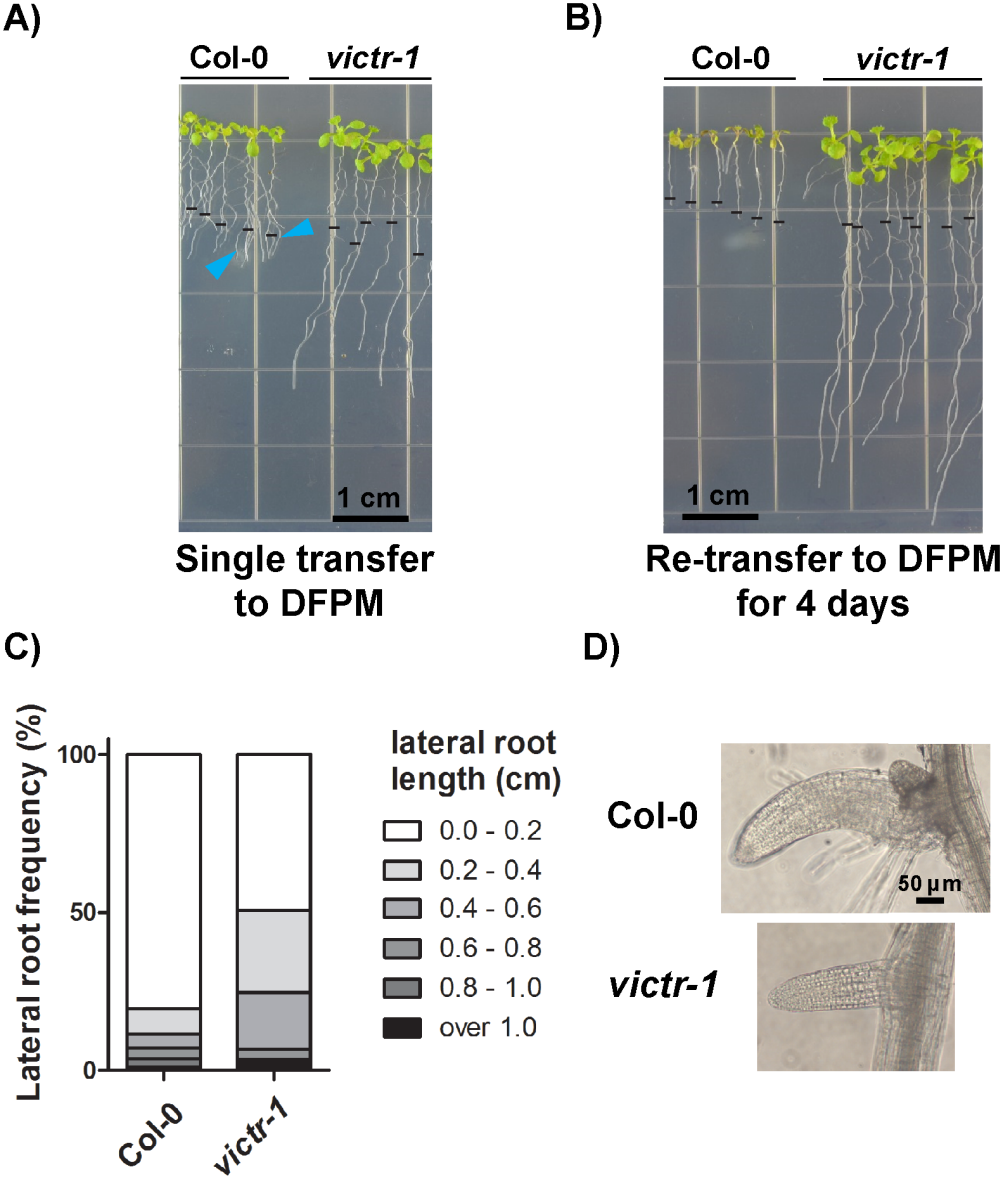
Growth arrest of lateral roots when Arabidopsis roots were exposed to fresh DFPM for a prolonged period. A) Transfer of wild-type Col-0 plants to DFPM medium one time inhibited primary root growth, while lateral roots could elongate further (arrowheads). Images were taken 7 days after the first transfer. DFPM-insensitive *victr-1* was used as a control. B) Prolonged exposure to fresh DFPM by re-transferring seedlings daily for 4 days resulted in further inhibition of growth in lateral roots and leaves in wild type (Col-0). Wild type (Col-0) and *victr-1* seedlings were transferred to 10 μM DFPM or DMSO control medium one time (A) or every 24 hours for 4 days (B). The black horizontal bars indicate the primary root lengths at the time of first transfer to DFPM. Primary and lateral roots of *victr-1* grew normally in all cases similarly to non-treated controls. C) Lengths of Col-0 and *victr-1* lateral roots in B were measured and shown as frequency distribution. n = 2, 12–19 plants per experiment. D) Lateral roots of Col-0 plants in (B) were swollen and bent similar to typical symptoms for primary roots treated with DFPM [15]. Scale bar applies for both images.

The light-dependent DFPM conversion raised the question after DFPM loses its yellow color in the presence of light, whether this colorless DFPM derived solution would maintain its bioactivity. To test this hypothesis, Col-0 wild-type Arabidopsis seedlings were exposed to 10 μM DFPM containing plates in the light. After 24 hours when the DFPM growth medium became colorless, one half of the seedlings were left on the same DFPM medium plates (Fig 5A) and the other half was re-transferred to a fresh DFPM medium. Re-transfer was repeated every 24 hours for four consecutive days (Fig 5B). Images were taken 7 days after the first transfer for both re-transfer and single transfer groups. The condition with the single transfer to the DFPM medium (Fig 5A) reflects the previously used assay [15]. While both DFPM conditions mediated irreversible primary root growth arrest, lateral root growth as well as shoot growth was further strongly compromised in the re-transfer group (Fig 5B). DFPM-tolerant *victr* plants maintained growth of primary roots in both experiments (Fig 5A and 5B). These data indicate that, although light exposure is required for DFPM bioactivity in root tissues (Fig 4), prolonged exposure to light in DFPM-containing MS growth media seems to reduce DFPM bioactivity eventually.

As previously described [15] under the single DFPM treatment, DFPM-triggered growth arrest is observed in primary roots of Col-0 wild-type seedlings, while lateral roots elongated even longer than arrested primary roots (Fig 5A, arrowheads). However, when seedlings were re-transferred to a fresh DFPM plate every 24 hours for 4 days, secondary root growth was also severely inhibited as well as the primary root growth (Fig 5B and 5C). *victr-1* mutant plants did not show growth arrest of either primary or lateral roots under all conditions of DFPM application (Fig 5). The tips of compromised Col-0 lateral roots in Fig 5B became swollen and bent into a hook-shaped structure (Fig 5D), resembling primary roots exposed to DFPM [15]. These observations suggest that DFPM effects are not limited to primary root growth arrest as assumed earlier [15] but also inhibit lateral root growth.

### Modification of DFPM in the presence of light and O_2_

**Fig 6:**
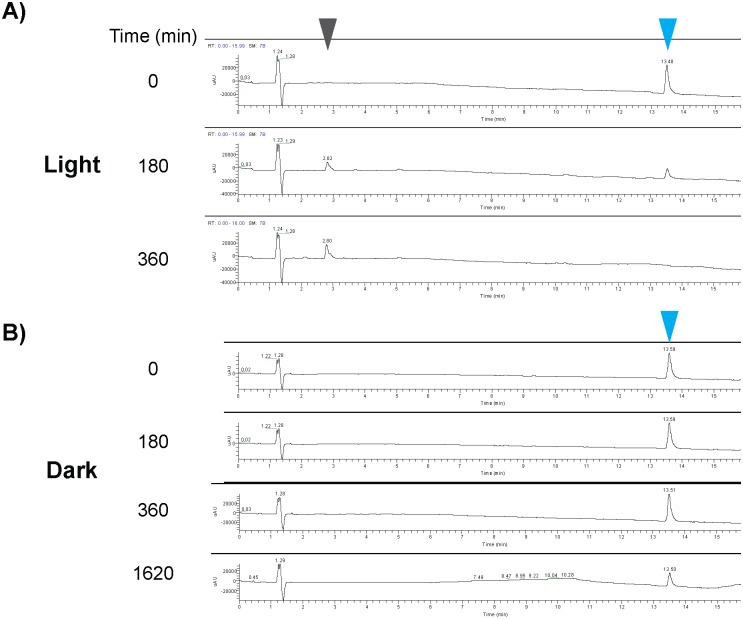
LC-MS analyses of DFPM modification in the presence or absence of light. DFPM in aqueous solutions gave a distinct absorption peak in non-treated control sample (t = 0) (λ_max_ = 318 nm, blue arrowheads). A) This peak declined after 180 and 360 min DFPM solution incubation in the light. At the same time a second peak was detected which accumulated over time in the light (grey arrowhead). B) If the same solution was incubated in the dark, the DFPM peak continued to decline but more slowly over time (blue arrowheads). No secondary metabolite was detected in the absence of light (B).

In an effort to gain insights into the observed DFPM modification process (Fig 4), the components of an aqueous DFPM solution were monitored by liquid chromatography-mass spectrometry (LC-MS) after being exposed to light over a period of 0, 180, and 360 min. In general, DFPM ionized well in electrospray ionization (ESI) and exhibited a distinct relative absorption peak with the retention time of around 13.5 min (Fig 6, blue arrowheads) where the wavelength of maximum absorption observed (λ_max_) was 318 nm. This distinct peak allowed for relative quantification of DFPM at defined time points. Consistent with earlier UV-Vis spectra (Fig 4B), after incubation in the light condition at room temperature for only 180 minutes a significant reduction in the characteristic peak of DFPM was detected (Fig 6A). After incubation for 360 min, the DFPM peak, both in the UV and extracted ion chromatogram (EIC), was completely absent (Figs 4B and 6A). Interestingly, as the characteristic peak of DFPM disappeared from the solution, a corresponding new peak appeared at a much shorter retention time of around 2.8 min (Fig 6A, grey arrowhead), indicating the formation of a new compound with increased polarity. This new peak whose λ_max_ is 257 nm exhibited poor ionization in ESI, and also had a different UV chromatogram, compared to DFPM. However, when the DFPM working solution was stored in the dark, no noticeable decrease of the DFPM peak (Fig 6B, blue arrowhead) was observed after incubation for 360 min, and the aforementioned more polar peak was not detected, even after incubation times as long as 1620 minutes (Fig 6B).

The effect of molecular oxygen on the modification process of DFPM was evaluated by creating oxygen enriched and depleted environments in aqueous solutions of DFPM. In the samples enriched in O_2_ (gas), acceleration in the degradation of DFPM was observed as well as the production of the unknown polar metabolite (S3A Fig). DFPM was at the detection limit of the LC-MS after only 180 min and undetectable at 360 min. In dark-treated samples that were also enriched with O_2_ (gas), no strong decrease of the DFPM peak or production of the polar metabolite was observed after 360 min (S3C Fig). However, due to the low solubility of DFPM in aqueous buffers, DFPM precipitated from the solution over time, also explaining the loss in absorbance in the UV-Vis spectrum (Fig 4B). These results suggest that both light and O_2_ are necessary for the modification of DFPM.

DFPM-7 represents a closely related DFPM derivative that completely lacks bioactivity [14, 15]. The chlorination pattern on the phenyl ring of DFPM-7 is different from DFPM, where DFPM-7 has a 2,5-dichlorophenyl versus the 3,4-dichlorophenyl in DFPM (S4 Fig). A shifted position of chlorine compared to DFPM seems critical to the lack of ability of DFPM-7 to elicit root growth arrest [15]. Therefore, DFPM-7 was included as a control to evaluate the stability of DFPM-7 under the same conditions. Although DFPM-7 decomposed in aqueous solution and lost its yellow color on a similar time scale as DFPM, there was no detectable accumulation of a derived product (S4 Fig Compare with the grey arrowhead in Fig 6). As observed for DFPM also, dark-treated DFPM-7 was found to be stable as revealed by its persistent HPLC peak (S4 Fig).

The absence of a derived product both in the DFPM solution that was incubated in the dark and in the DFPM-7 solutions prompted additional investigations to identify the unknown substance (grey arrowhead in Fig 6), as it may be responsible for the observed bioactivity in the root growth assay. High-resolution atmospheric pressure chemical ionization mass spectrometry (HR-APCIMS) analyses of the modified product of DFPM gave a [M+H]^+^ at *m/z* 340.0496, indicating a molecular formula (MF) of C_16_H_15_Cl_2_NO_3_ (Δ 1.8 ppm). Interestingly, it only varies from DFPM (MF C_16_H_15_Cl_2_NOS) by the addition of O_2_ and loss of sulfur. Furthermore, comparison of the UV data for DFPM and this modified product revealed significant modification in the conjugated double bond system of DFPM as there was a shift in absorption from 318 to 257 nm, respectively. Based on these findings and the increased polarity of the modified product compared to DFPM, we propose a potential structure and mechanism for this modified product (S5 Fig). According to our model, molecular oxygen reacts with the furan ring in DFPM in a light-induced Diels-Alder type reaction [26], forming an endoperoxide. This peroxide may then degrade, similarly to that involved in ozonolysis of an olefin [27], forming the compound presented in S5 Fig. All attempts to isolate and enrich this modified product to test its bioactivity by pursuing root bioassays on plant seedlings failed, likely as a result of the compound’s inherent instability.

## Discussion

### New *victr* alleles reveal important residues for R-protein-dependent signaling

The DFPM-mediated root growth response is dependent on a Col-0 accession-specific TIR-NB-LRR locus named *VICTR* [15]. In the initial discovery of this functional relationship, it remained uncertain whether *VICTR* is a canonical *R-*gene which requires a functional nucleotide binding domain. Here, new *victr* alleles isolated by independent forward chemical genetic screening confirm the involvement of *VICTR* in DFPM signaling. The identification of the first independent *victr* EMS point mutant lines now gives further insights into VICTR function. Most *R*-genes require binding of ATP/ADP and hydrolysis for full activation [28]. Because the recessive mutation in *victr-6* results in a glycine 216 to Asp mutation in the conserved Walker A, or P-loop, motif (*GX*_*4*_*GK*[*T/S*]) (Fig 1), it is probable that loss of the highly conserved glycine in *victr-6* diminishes ATP/ADP binding and hydrolysis to the NB domain. The conserved Walker A motif (*GX*_*4*_*GK*[*T/S*]) was shown in earlier studies of *R*-gene function to be critical for disease resistance [18, 19, 29]. The two mutations identified in the C terminus of the *victr-7* and *victr-8* protein variants are less conserved among R-proteins and therefore might be required for a more specific VICTR function, such as binding a ligand or other protein. However, it is not known whether these mutations affect VICTR steady-state protein accumulation. The presented findings support a requirement for the VICTR conserved Walker A domain and putative ATP/ADP binding and hydrolysis activity in the DFPM-mediated immune response.

Notably, exposure to DFPM was found to substantially increase EDS1-promoter driven EDS1-YFP protein fluorescence in roots (Fig 2, S1 Fig). This observation correlates with activation of a positive feedback loop on *EDS1* expression in ETI leaf responses [30]. The striking difference in YFP fluorescence between non-induced and DFPM-activated root cells may enable additional spatial analyses of VICTR-dependent EDS1 signaling dynamics in the root.

### Nuclear and cytoplasmically-enriched EDS1 pools are competent in DFPM-induced root growth arrest

Nucleo-cytoplasmic EDS1 and its interacting partner PAD4 are central modulators of basal resistance to virulent pathogens and TIR-NB-LRR mediated ETI [3]. EDS1 resides in complexes with well characterized TIR-NB-LRR RPS4 and RPS6 as well as VICTR [15, 23, 31]. However, a complete picture of the molecular signaling mechanisms remains to be established. Evidence from EDS1 subcellular targeting experiments indicated an essential nuclear activity of EDS1, likely with nuclear PAD4, in basal immunity and TIR-NB-LRR ETI in leaves [16, 17, 21]. A separate EDS1 immune signaling branch was proposed to promote cell death in ETI in the cytoplasm [17, 23]. Here, we tested whether EDS1 nuclear- or cytoplasmic distribution is important for TIR-NB-LRR (VICTR) dependent DFPM growth responses in roots. We found that transgenic expression of either NES- or NLS-tagged EDS1-YFP fully complemented the root growth arrest phenotype of *eds1-2* elicited by DFPM (Fig 2). These data suggest that nuclear or cytoplasmically-enriched EDS1 pools are competent in VICTR signaling. We cannot exclude that low amounts of nuclear EDS1 remaining in nuclei when EDS1 protein export is enhanced, are sufficient to signal in VICTR root responses [17, 21]. Indeed, characterization of EDS1-YFP-NLS line #B2 which expresses low EDS1-YFP protein accumulation [21] (S2 Fig) indicates that a low level of nuclear-targeted EDS1 is sufficient to signal pathogen resistance in leaf cells [21]. It is therefore conceivable that DFPM-induced VICTR signaling leading to root growth arrest requires only a small pool of nuclear EDS1.

VICTR, the only presently known TIR-NB-LRR involved in DFPM signaling, can form protein complexes with both EDS1 and PAD4, and their protein complexes were found mostly in the nucleus [15]. Nuclear localization of this R-protein-EDS1 complex might therefore be different from other Arabidopsis R-protein-EDS1 complexes such as RPS4 and RPS6 that were found in the cytoplasm and the nucleus [23, 31]. Moreover, VICTR, but not RPS4 or RPS6, was detected in a complex with PAD4 [15, 31]. The mainly nuclear localization VICTR complexes with EDS1 or PAD4 suggest that DFPM-mediated VICTR signaling immediately engages EDS1 and PAD4 to regulate downstream target pathways. In line with this, we found that *VICTR* gene expression is to a large extent EDS1-dependent (Fig 2, S1A Fig). Several studies indicate that EDS1 and PAD4 contribute to defense responses to various degrees depending on the pathogen, pest or effector types [32–34]. Rietz et al. (2011) showed that EDS1 and PAD4 are both necessary for a cell death response to avirulent *Hyaloperonospora arabidopsidis*. In contrast, *PAD4* is dispensable for *RPS4*/*RRS1* resistance to the avirulent *Pseudomonas syringae* DC3000/avrRps4 strain because its paralog, *SAG101*, can compensate for loss of *PAD4* [6,32,35]. Open questions for DFPM-activated R-protein signaling include whether other factors that can be shuttled between the nucleus and the cytoplasm are involved. The DFPM-mediated root growth arrest provides a powerful system and read-out for genetic dissection of additional and new components that function in R-protein signaling.

### DFPM becomes bioactive during light and oxygen-dependent modification

DFPM was isolated from a large-scale chemical library screen [14]. Here we show that DFPM has limited lifetime and loses its yellow color in non-DMSO solutions especially in the presence of light (Fig 4). Six hours of light exposure were sufficient to decrease the DFPM peak below the detection limit by LC-MS (Fig 6). Interestingly, the original DFPM compound is not bioactive in arresting root growth when protected from the light, but becomes bioactive when modification is initiated by light exposure (Fig 4C). Our chemical analyses suggest that the product of DFPM modification with a molecular ion peak of 340.0496 *m/z*, or possibly related byproducts, are most likely responsible for the observed bioactivity (S5 Fig, see the gray arrowhead in Fig 6A). First, this product is observed by LC-MS only in the presence of light and O_2_, and not in the dark or under O_2_-depleted conditions (Fig 6, S3 Fig). Second, a non-functional structural analog, DFPM-7, whose chemical structure only varies in the position of chlorine [14] did not yield a comparable product by LC-MS (S4 Fig). Moreover, the present findings suggest that this metabolite degrades further, since the bioactivity of the DFPM solution decreases eventually after it loses its yellow color by a prolonged exposure to light (Fig 5A and 5B). This circumstance prevented direct bioassays using the compound since purification and enrichment were unsuccessful. Further investigations will be needed to confirm the precise nature of the bioactive DFPM modified product. Nevertheless, the present study predicts a possible structure that could represent this modified product (S5 Fig).

In conclusion, the present study supports a canonical R-protein function of VICTR in roots, and shows that VICTR-dependent DFPM-induced root growth arrest occurs irrespective of whether EDS1 is targeted to the nuclear or cytoplasmic compartments. We further demonstrate the potential of DFPM-mediated root growth arrest for dissecting an R-protein-mediated signaling pathway in roots and report on optimized handling of DFPM for chemical genetic screening of R-protein-dependent signaling components.

## Material and Methods

### Plant materials and chemicals

*Arabidopsis thaliana* mutants *victr-1* (Salk_123918) and *eds1-2* (in Col-0 accession) were described in Kim et al. [15]. All EDS1-YFP transgenic lines were generated in *eds1-2* (Col-0 accession). EDS1-YFP, EDS1-YFP-NES and EDS1-YFP-nes transgenic lines are described in Garcia et al. [17], and EDS1-YFP-NLS transgenic lines are described in Stuttmann et al. [21]. DFPM ([5-(3,4-dichlorophenyl)furan-2-yl]-piperidine-1-ylmethanethione) was purchased from Chembridge (chemical ID 6015316) and Innovapharm (Ukraine, chemical ID STT-00334837).

### DFPM root growth assay and subcellular localization of EDS1 isoforms in the root tissue

As described before [15], seeds were sterilized and germinated on half MS medium (half-strength Murashige and Skoog Basal Medium with Gamborg′s vitamins (M0404 Sigma), 0.05% MES, pH 5.7, 1% sucrose) plates with 0.8% phyto agar. After 2 days of stratification, seedlings were vertically grown for 10 days in 16 h/8 h long day conditions. On day 10, plants were transferred to new plates containing 10 μM DFPM. Subsequently, the root tip position was marked and root growth arrest was monitored after 6 d. All root growth experiments were repeated at least three times in independent experiments and representative images are shown.

For subcellular localization studies of EDS1-YFP isoforms within root cells, 5 day-old plant seedlings were exposed to 10 μM DFPM for 24 h and monitored using a Zeiss LSM 710 confocal microscope (Ex 514 nm/ Em 519–621 nm). Constant gain and pinhole values were used to observe pEDS1-EDS1-YFP, pEDS1-EDS1-YFP-NES and pEDS1-EDS1-YFP-NLS high expressor #A5. For pEDS1-EDS1-YFP-nes and the pEDS1-EDS1-YFP-NLS low expressor line #B2, an increased gain value was used to enhance the YFP signal.

### Quantitative Real-time RT-PCR

Plants were grown in 1/2 MS plates with 1% sucrose for 14 days and transferred to a 24 well plate containing 1/2 MS solution with 10 μM DFPM or DMSO for 24 hours. Total RNA was extracted using Spectrum^™^ Plant Total RNA Kit (Sigma-Aldrich), treated with TURBO^™^ DNase (Thermo-Fisher) and reverse-transcribed using First-Strand cDNA Synthesis Kit (GE Healthcare). Real-time RT-PCR was performed to quantify *VICTR*, *PR5* or *EDS1* transcript levels. Amplified samples were normalized against the housekeeping gene *PDF2*. To extract RNA from shoots and roots separately, plants were grown in vertical plates. After DFPM treatment, shoots and roots were isolated and used for RNA extraction separately as mentioned earlier. Primers used are: VICTR-qRT2-F 5’- AGAGACCGGTTCATCAGCAGAG-3’, VICTR-qRT2-R 5’-CCATATTGCCTTCTTCGGCTTGAG-3’, PR5-F 5’-ATCACCCACAGCACAGAGACAC-3’, PR5-R 5’-AGCAATGCCGCTTGTGATGAAC-3’, EDS1 qRT-F 5’-GCTCAATGACCTTGGAGTGAGC-3’, EDS1 qRT-R 5’-TCTTCCTCTAATGCAGCTTGAACG-3’, PDF2-F 5’-TAACGTGGCCAAAATGATGC-3’, PDF2-R 5’-GTTCTCCACAACCGCTTGGT-3’.

### FDA cell viability assay using microplate reader

Half-strength MS medium was aliquoted into a white 96 well plate (08-771-26 Corning Life Sciences). Subsequently, one sterilized seed per well was added and the plate sealed using parafilm. After 2 d of stratification seedlings were grown in a Percival growth cabinet (150 μmoles/m²/s) for 7 d in 16 h/8 h long day conditions. After 7 days, 130 μL of the same media as listed above (- agar), +/- DFPM was added to each well (15 μM DFPM final concentration). Half of the plate was wrapped with aluminum foil to protect the samples from light. Plates were returned to the Percival growth chamber for a 6 h incubation time. From a 250 μg/ml fluorescein diacetate (FDA) stock (Sigma, F7378, in DMSO) 5.5 μL/well were added to get a final concentration of ~5 μg/ml. After 5 min incubation at room temperature, cell viability was determined by fluorescence detection (Ex 485 nm /Em 510 nm) using a Berthold plate reader (Mithras LB 940).

### FDA cell viability assay in roots

Seven day-old seedlings were incubated in half MS medium containing DFPM at the indicated concentrations for 24 hours. Seedlings were stained with 7 μg/ml FDA and 5 μg/ml propidium iodide (Sigma, P4170) for 10 seconds. After a brief rinse with water, root cells were monitored using a Zeiss LSM 710 confocal microscope (Ex 488 nm/ Em 493–555 nm). Constant gain and pinhole values were used to observe fluorescence.

To quantify FDA fluorescence signals and calculate IC_50_ values, seedlings exposed to DFPM with increasing concentrations from 0.5 to 10 μM were stained with 7 μg/ml FDA and observed using a Nikon Eclipse TE2000-U Confocal microscope (Ex 488 nm/ Em 500–550 nm). Fluorescence intensity was measured using Image J program [36] and IC_50_ was measured using Graphpad Prism 6 program.

### Methods for examining degradation products of DFPM

To obtain aqueous working solutions for all experiments, 7.5 μL of a 50 mM DFPM stock solution in DMSO was added to 5 mL of MilliQ water in a glass vial (75 μM final conc.). The solutions were thoroughly mixed and 100 μL was used for time zero control (T0) Liquid Chromatography–Mass Spectrometry (LC-MS) analyses. For light reactions, the vial was then placed, uncapped, in front of a UV lamp. For the dark reactions, the vials were immediately covered in aluminum foil and placed right next to the light reaction samples, uncapped. For the experiments with either O_2_ (gas) enrichment or depletion, upon addition of DFPM the vials were capped with a rubber septum and attached to a vacuum manifold. Using the manifold the air in the vial was replaced with either argon gas (O_2_ depletion) or O_2_ (gas) (O_2_ enrichment) and then placed in front of the UV light. To ensure constantly remaining O_2_ (gas) levels in the enriched samples, a balloon filled with O_2_ (gas) adapted with a needle was inserted through the rubber septum during the entire experiment. At every given time point, 100 μL sample aliquots were removed for LC-MS analyses. The LC-MS analyses were performed using a ThermoFinnigan Surveyor, equipped with a C18 Kinetic core-shell column. Solvent system used: holding 50% acetonitrile (ACN)/H_2_O + 0.1% formic acid (FA) for 3 min, then gradient to 100% ACN over 12 min, and concluding with a 5 min hold at 100% ACN. High resolution mass spectra were acquired for both DFPM and the modification product using an Agilent 6230 LC-APCI-TOFMS equipped with the same column and gradient as described above.

## Supporting Information

S1 Fig DFPM-mediated transcriptional induction of *VICTR* and *EDS1*. A) DFPM induction of *VICTR* and pathogen response marker gene *PR5* mRNA levels is impaired in *eds1-2* mutant and is partially dependent on *EDS1*. Expression of *VICTR* and *PR5* was increased by 10 μM DFPM in Col-0 wild type and to a lesser extent in *eds1-2*. Data from two independent quantitative RT-PCR experiments are shown. B) *EDS1* gene expression was induced in both shoot and root tissues in response to DFPM. Col-0 wild type plants were treated with 3 μM or 10 μM DFPM and *EDS1* transcript levels were determined in shoot or root tissues by qRT-PCR.
(TIF)

S2 Fig EDS1-YFP localization in roots of EDS1-YFP-nes #1–2 (A) and EDS1-YFP-NLS #B2 (B) lines in *eds1-2*. All EDS1-YFP signals increased after 24 hours of DFPM application compared to the non-treated control. Scale bar applies to all 4 images. Constant gain and pinhole parameters were used for all 4 images.
(TIF)

S3 Fig DFPM modification is O_2_ dependent. A) When DFPM was exposed to an enriched O_2_ environment, modification was accelerated compared to ambient O_2_ levels (blue arrowheads) and a new metabolite peak appeared (grey arrowhead). B) When O_2_ was depleted from the solution only a slow DFPM precipitation was detected. C-D) No DFPM metabolite peak (grey arrowhead) was detected in the dark. O_2_ level had only minor effects in the dark-treated DFPM solution.
(TIF)

S4 Fig DFPM-7 modification differs from DFPM modification. A) Chemical structures of DFPM and DFPM-7 [14]. DFPM has a 3,4-dichlorophenyl while DFPM-7 has a 2,5-dichlorophenyl. B) If the non-bioactive derivative DFPM-7 in aqueous solutions was exposed to light, precipitation over time occurred but no metabolite peak was detected (See Fig 6 and S3 Fig). C) In the absence of light the DFPM-7 peak (blue arrowhead) was relatively stable at 360 min.
(TIF)

S5 Fig Proposed reaction mechanism to yield the modified DFPM metabolite (*m/z* 340.0496, molecular formula of C_16_H_15_Cl_2_NO_3_). The furan ring in DFPM may react with O_2_ in a light-induced Diels-Alder type reaction, forming an endoperoxide. This peroxide may degrade similarly to that involved in ozonolysis of an olefin (See text for details).
(TIF)
