# Supplementary figures and images for: Small Molecule DFPM Derivative-Activated Plant Resistance Protein Signaling in Roots Is Unaffected by EDS1 Subcellular Targeting Signal and Chemical Genetic Isolation of victr R-Protein Mutants
Source: PLoS One. 2016 May 24;11(5):e0155937. doi: 10.1371/journal.pone.0155937 (PMC4878808; doi:10.1371/journal.pone.0155937)

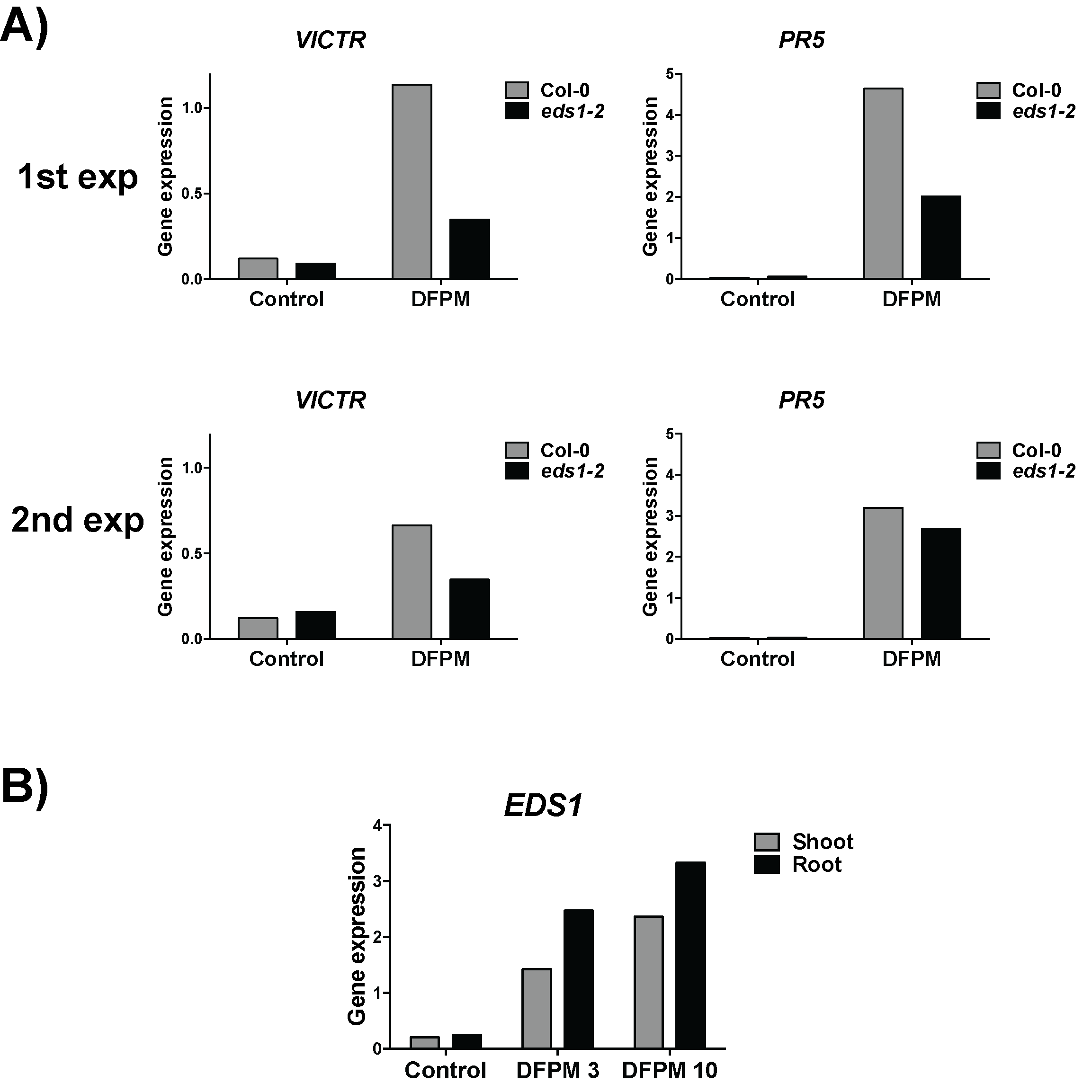

Supplement: S1 Fig — A) DFPM induction of VICTR and pathogen response marker gene PR5 mRNA levels is impaired in eds1-2 mutant and is partially dependent on EDS1. Expression of VICTR and PR5 was increased by 10 μM DFPM in Col-0 wild type and to a lesser extent in eds1-2. Data from two independent quantitative RT-PCR experiments are shown. B) EDS1 gene expression was induced in both shoot and root tissues in response to DFPM. Col-0 wild type plants were treated with 3 μM or 10 μM DFPM and EDS1 transcript levels were determined in shoot or root tissues by qRT-PCR. (TIF) [file pone.0155937.s001.tif]

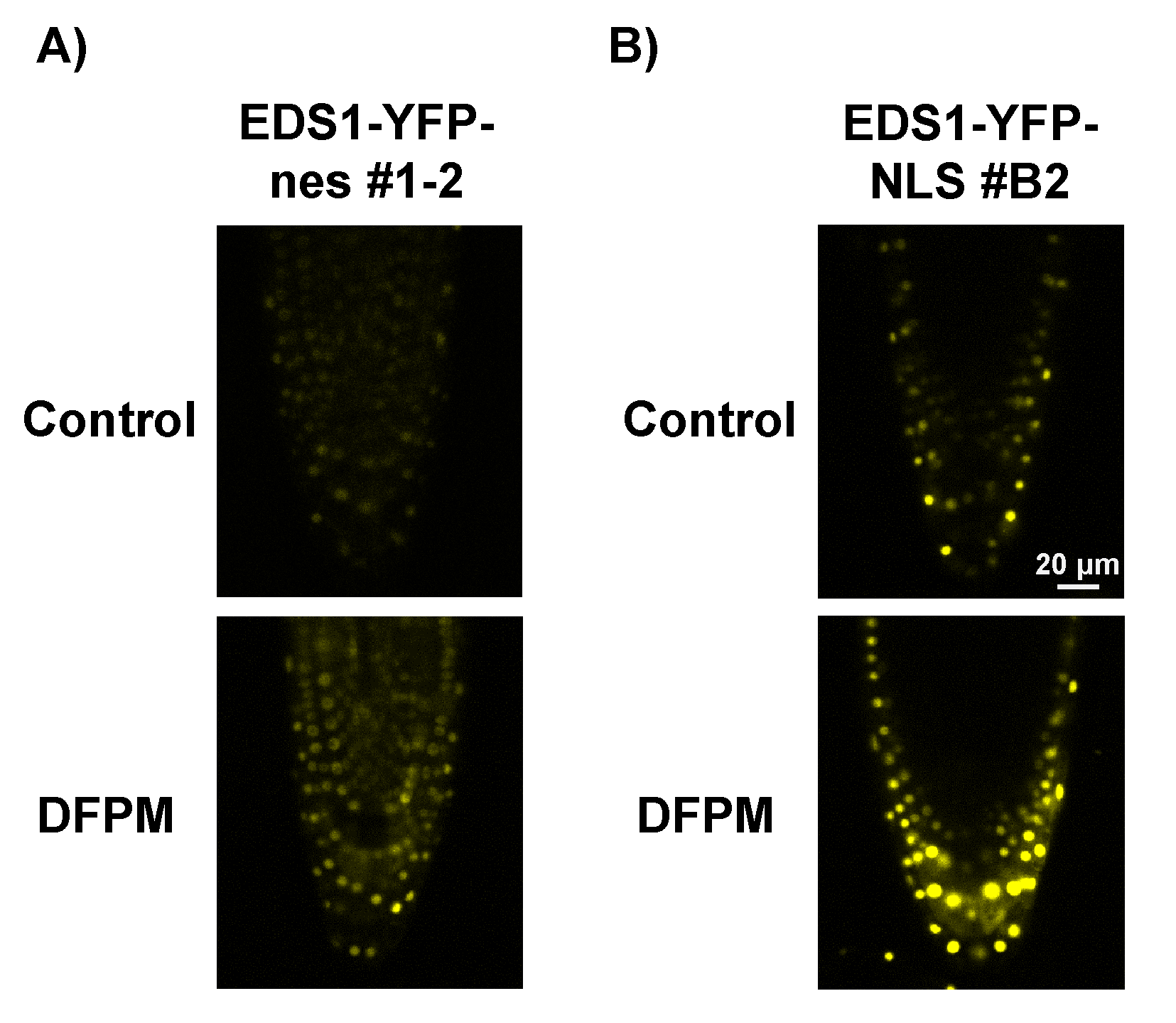

Supplement: S2 Fig — All EDS1-YFP signals increased after 24 hours of DFPM application compared to the non-treated control. Scale bar applies to all 4 images. Constant gain and pinhole parameters were used for all 4 images. (TIF) [file pone.0155937.s002.tif]

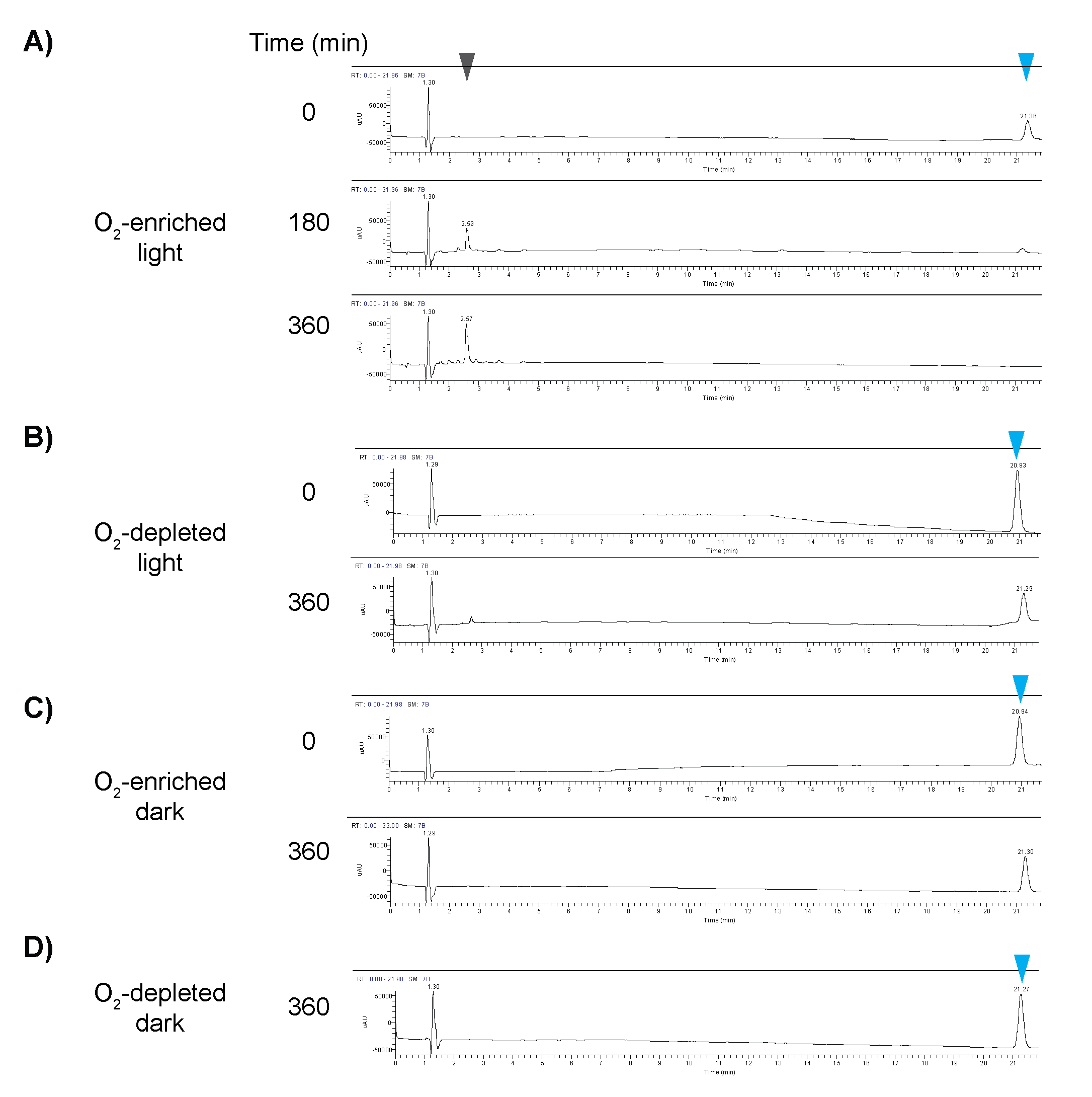

Supplement: S3 Fig — A) When DFPM was exposed to an enriched O2 environment, modification was accelerated compared to ambient O2 levels (blue arrowheads) and a new metabolite peak appeared (grey arrowhead). B) When O2 was depleted from the solution only a slow DFPM precipitation was detected. C-D) No DFPM metabolite peak (grey arrowhead) was detected in the dark. O2 level had only minor effects in the dark-treated DFPM solution. (TIF) [file pone.0155937.s003.tif]

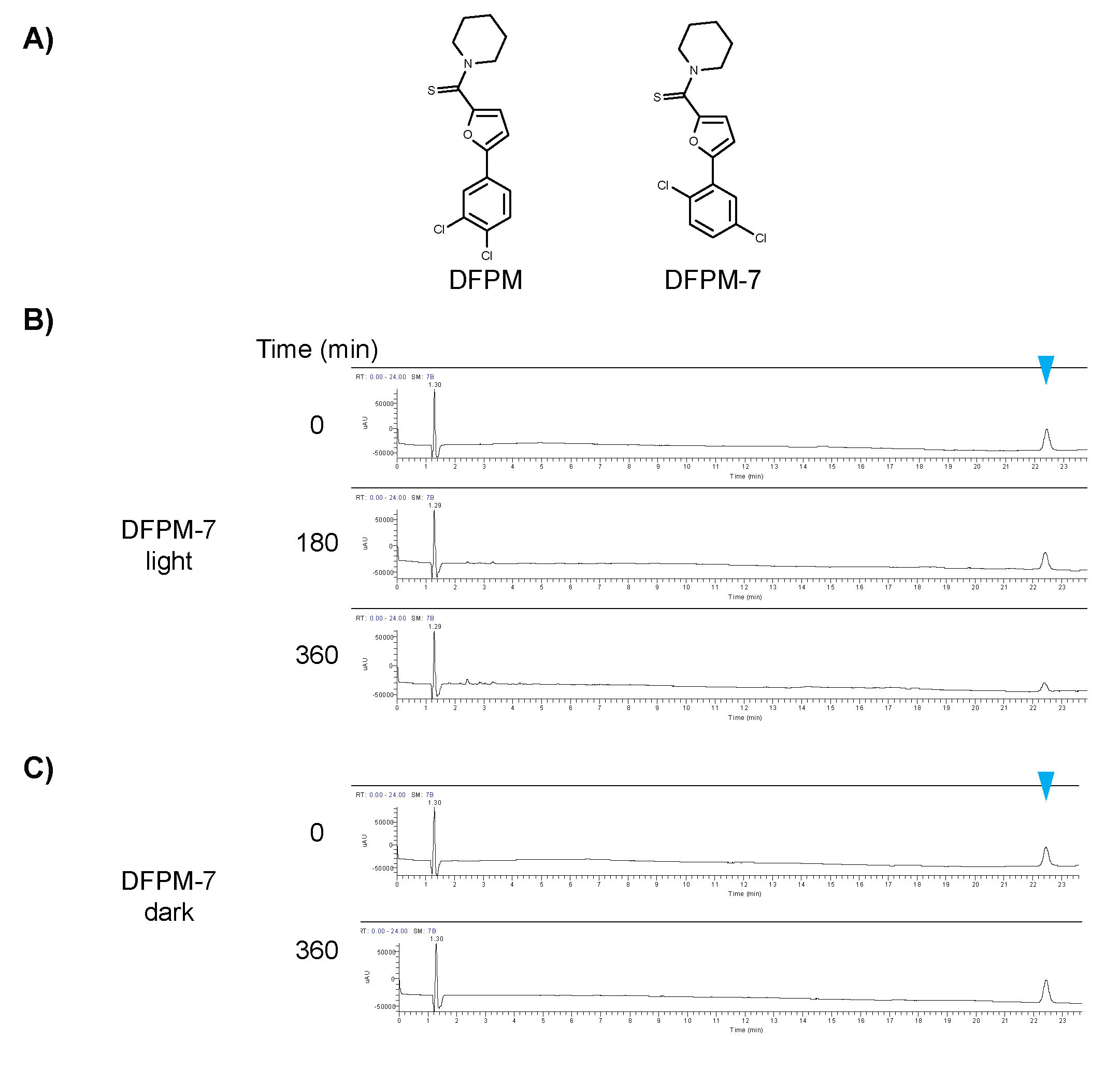

Supplement: S4 Fig — A) Chemical structures of DFPM and DFPM-7 [14]. DFPM has a 3,4-dichlorophenyl while DFPM-7 has a 2,5-dichlorophenyl. B) If the non-bioactive derivative DFPM-7 in aqueous solutions was exposed to light, precipitation over time occurred but no metabolite peak was detected (See Fig 6 and S3 Fig). C) In the absence of light the DFPM-7 peak (blue arrowhead) was relatively stable at 360 min. (TIF) [file pone.0155937.s004.tif]

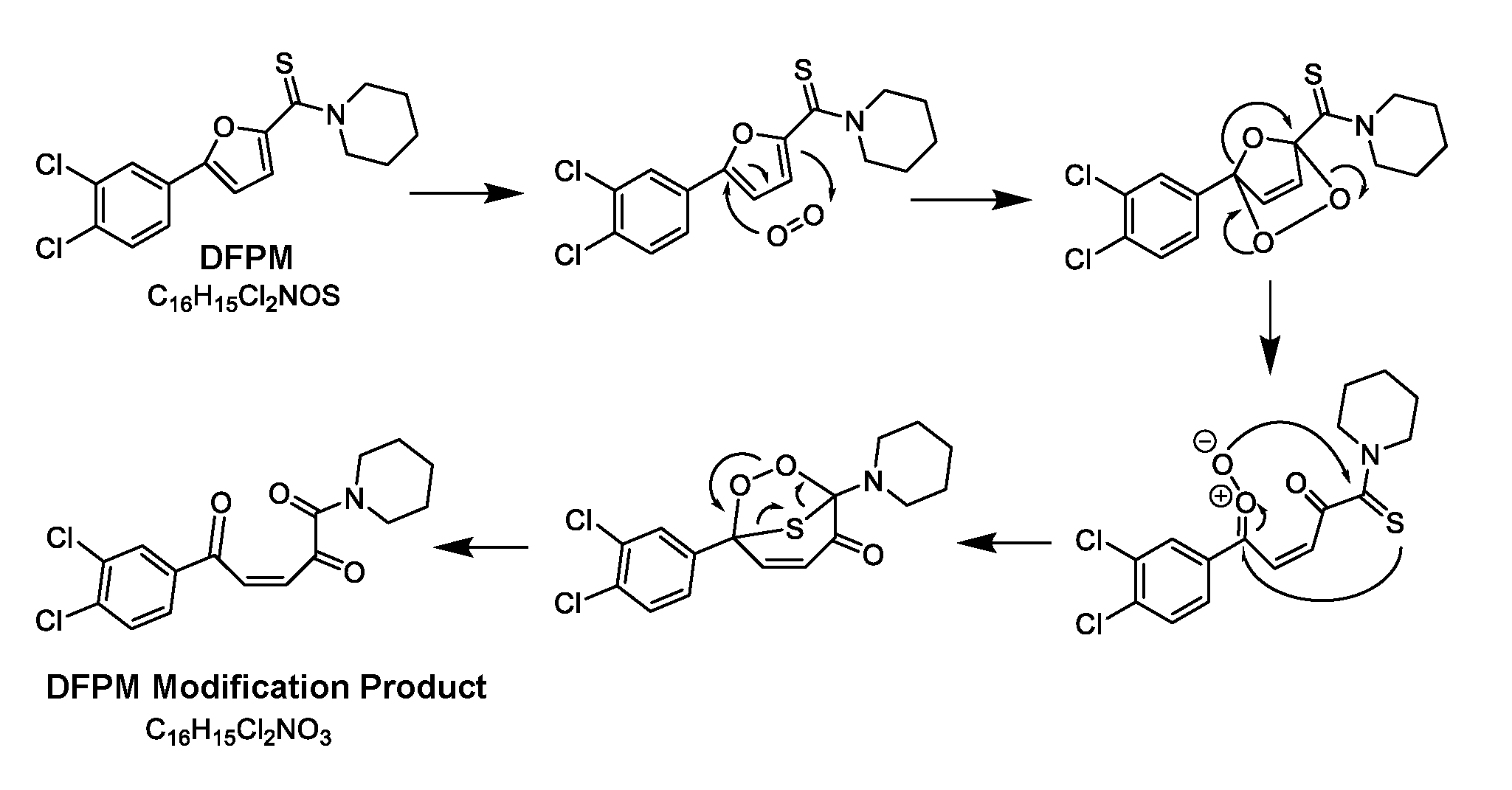

Supplement: S5 Fig — The furan ring in DFPM may react with O2 in a light-induced Diels-Alder type reaction, forming an endoperoxide. This peroxide may degrade similarly to that involved in ozonolysis of an olefin (See text for details). (TIF) [file pone.0155937.s005.tif]
